# HTLV-1 and bronchiectasis in a UK cohort, report and review

**DOI:** 10.1186/1742-4690-11-S1-P11

**Published:** 2014-01-07

**Authors:** Shohreh Honarbakhsh, Graham P Taylor

**Affiliations:** 1 National Centre for Human Retrovirology, St Mary’s Hospital, Imperial College Healthcare NHS Trust, London, UK; 2 Imperial College London, National Centre for Human Retrovirology, St Mary’s Hospital, Imperial College Healthcare NHS Trust, London, UK

## Background

Associations between HTLV-1 and pulmonary disease have been reported but causality and risk have not been confirmed. Pulmonary function tests have been routinely offered to new patients attending the UK HTLV service whilst a retrospective case review has been conducted to determine the prevalence of diagnosed bronchiectasis.

## Method

The cohort was categorised into HTLV-1 symptomatic patients (SPs) (ATLL, HAM/TSP, polymyositis and strongyloidiasis) and asymptomatic carriers (ACs). Computerised tomographic (CT) imaging performed was reviewed. In 60 patients disease state was correlated with pulmonary function and HTLV-1 proviral load (VL).

## Results

8/249 ACs and 27/164 SPs had a CT, with productive cough +/- recurrent chest infections the predominant indication. Bronchiectasis was diagnosed in one AC (1/249) and 10 SPs (1 polymyositis, 1 ATLL, 8 HAM/TSP) (10/164, OR 16.10; p=0.0084). In univariate analysis increased rates of bronchiectasis were seen in HAM/TSP patients compared with all other categories (OR 14.1 p<0.0001) and non-African/Afro-Caribbean ethnicity subjects (OR 4.2; p=0.019) whilst age was significantly associated with bronchiectasis (p=0.002). PEFR (4.96 l/s vs. 6.77 l/s; p=0.0003), FEV1 (2.22 vs. 2.57; p=0.06) and KCO (1.35 s^-1^ vs. 1.59 s^-1^; p=0.0029) were all lower in SPs (n-27) compared with ACs (n-33) with a negative correlation between VL and PEFR (rr-0.25).

## Conclusion

Bronchiectasis was common in the cohort and therefore HTLV serology should be considered in patients with bronchiectasis. Patients with non-pulmonary HTLV-1-associated disease are more likely to have an additional diagnosis of bronchiectasis and obstructive lung disease pattern than ACs suggesting an inflammatory aetiology.

